# Effective interventions to prevent catheter-associated urinary tract infections: a systematic review

**DOI:** 10.1590/S1678-9946202567061

**Published:** 2025-10-03

**Authors:** Na Li, Rongjie Shi, Yan Sun, Qingli Chen

**Affiliations:** 1 The First Affiliated Hospital of Nanjing Medical University, Jiangsu, Nanjing, China

**Keywords:** Catheter, Urinary tract infections, Ureteral catheter, Catheter care protocol, Intervention

## Abstract

Catheter-associated urinary tract infections (CAUTIs) are a prevalent and preventable healthcare-associated infection that significantly impacts healthcare systems, contributing to increased patient morbidity, length of stay, and costs. This systematic review aims to evaluate the effectiveness of various interventions in reducing CAUTI incidence in healthcare settings. Following the PRISMA guidelines, we conducted a thorough literature search across multiple databases—Web of Science, Scopus, PubMed, MEDLINE, EMBASE, Cochrane Central Register of Controlled Trials, and Google Scholar—up to August 8, 2024. Eligible studies included randomized controlled trials, case-control studies, and experimental designs that met inclusion criteria, namely CAUTIs prevention among hospitalized adult patients. After screening 9,476 titles and abstracts, we reviewed 163 texts in full. Of these, 12 studies were included in this review. Results showed that antiseptic solutions like chlorhexidine, specialized catheters (e.g., BIP Foley and silver alloy-coated types) and educational sessions all significantly reduced CAUTI rates, with some interventions achieving reductions as high as 94%. Reminder systems promoting timely catheter removal and amikacin bladder washing also showed notable effectiveness. Adverse effects were minimal. This review underscores the importance of evidence-based CAUTI prevention strategies and the need for consistent implementation across healthcare facilities. Enhanced catheter maintenance practices and judicious catheter use can significantly reduce CAUTI rates, thereby improving patient outcomes and reducing healthcare-associated costs. Future research should continue exploring diverse, context-specific interventions to address barriers to CAUTI prevention.

## INTRODUCTION

Urinary tract infections (UTIs) pose a substantial healthcare challenge, accounting for up to 40% of all nosocomial infections^1^. Among these, catheter-associated urinary tract infections (CAUTIs) are a common and preventable type of healthcare-associated infection (HAIs) in hospitalized patients^2,3^. CAUTIs can lead to serious complications, including bacteremia which affects approximately 20% of hospital-acquired bloodstream infections. CAUTIs incidence rate ranges from 3% to 8% per day, with a prevalence of about 8.5%^1^. Additionally, UTI-associated bacteremia has a mortality rate of approximately 10%^4^. Several factors contribute to CAUTIs development, including unnecessary catheter use, improper insertion technique, and prolonged catheterization. These practices can introduce bacteria into the urinary tract, leading to infection and potential systemic spread^5^. The impact of CAUTIs is far-reaching. Studies have linked CAUTIs to increased length of stay, higher healthcare costs, antibiotic use, and morbidity and mortality rates^6^. This underscores the urgent need for effective interventions to reduce the risk of these infections^7^.

Various strategies have been proposed to reduce the risk of CAUTIs^8^, including minimizing unnecessary catheter use, ensuring correct insertion and maintenance practices, and promptly removing urinary catheters^9,10^. Catheterization duration significantly influences the risk of bacteriuria. For each day a urinary catheter remains in place there is a 3%–8% risk of bacteriuria, and prolonged catheterization (>28 days) typically results in continuous bacteriuria^11^. Thus, minimizing catheter use duration and removing catheters as soon as they are no longer medically appropriate is essential. Daily evaluations to determine the need for continued catheterization and prompt removal are critical^12^. Alternative bladder drainage strategies such as clean intermittent catheterization should be considered^12^. For example, external options like condom catheters for men and recent alternatives for women are viable solutions recommended by the CDC^13^.

Catheter use reduction requires hospital-wide programs, including adequate staffing, staff training, and access to essential equipment^14^. Using electronic medical records (EMR) to track catheter insertion and removal dates, along with reminders for removal, is also important. One study showed that CAUTI awareness education, insertion and removal protocols, and the use of PureWick female incontinence devices significantly reduced CAUTI rates^15^. Similarly, another study found that a nurse-driven protocol for early urinary catheter removal, as part of a multimodal CAUTI intervention strategy, led to measurable decreases in both catheter utilization and CAUTI rates^16^. A national prevention program implementing these interventions resulted in a decrease in adjusted CAUTI rates from 2.40 to 2.05 per catheter day (p = 0.009), with catheter use in non-intensive care unit settings reducing from 20.1% to 18.8%^8^.

Given the economic burden and clinical complications of CAUTIs on healthcare systems, effective, evidence-based interventions are needed. Despite being largely preventable, CAUTIs remain prevalent due to unnecessary catheterization, improper insertion techniques, and prolonged catheter use without timely removal, highlighting the importance of ongoing assessment and prompt removal strategies. Research indicates that comprehensive preventive measures can significantly reduce CAUTI rates^8,17^, supporting the implementation of systematic, multidisciplinary interventions. Structured analysis is crucial to identify the most effective strategies. This systematic review provides a comprehensive, evidence-based analysis of intervention effectiveness to guide healthcare providers in adopting best practices. By analyzing multiple studies, it supports the development of targeted, high-impact strategies to reduce CAUTI incidence, improve patient outcomes, lower healthcare costs, and enhance hospital care quality. Thus, this study was conducted to evaluate interventions aimed at preventing catheter-associated urinary tract infections.

## MATERIALS AND METHODS

### Ethics

This systematic review complied with the established ethical standards for research synthesis. As no primary data collection involving human participants was undertaken, formal ethical approval was not required. The review adhered to principles of transparency, rigor, and integrity in line with the PRISMA guidelines.

### Eligibility criteria

This systematic review adhered to the PRISMA 2020 approach for Systematic Review (PRISMA-SR) guidelines^18^. We employed specific inclusion and exclusion criteria to identify relevant studies for analysis.

The primary objective of this systematic review was to assess the overall effectiveness of various interventions in reducing the incidence of catheter-associated urinary tract infections (CAUTI), focusing on comparing the efficacy of antimicrobial catheters, catheter maintenance protocols, and other preventive measures in healthcare settings.

We used the CDC criteria for CAUTI which is characterized by the presence of at least one of the following: fever (temperature > 38 °C), suprapubic tenderness, costovertebral angle pain or tenderness, and a positive urine culture with ≥ 105 CFU of a single organism^19^. These studies examined various interventions and practices aimed at CAUTI prevention, including improved hand hygiene, regular monitoring, cleaning the urethral meatus with sterile water, use of silicone catheters with closed drainage systems, daily assessment of catheter need, limiting indwelling catheters for approved indications only, shortening catheter duration, and providing patient education and placement of collection devices.

### Inclusion criteria

Controlled and uncontrolled before-after studies, interrupted time series analyses (with a control group), randomized controlled trials, quasi-experimental studies, cohort studies, case-control studies, cross-sectional studies, and quantitative or qualitative studies were eligible. These studies had to evaluate the effectiveness of CAUTI prevention interventions, involve adult patients hospitalized in intensive care units, and be published in English.

### Exclusion criteria

We excluded reviews, case series, letters, notes, conference abstracts, opinion articles, studies focused exclusively on non-catheter-related urinary tract infections, studies involving patients with conditions that contraindicate standard catheterization practices or where catheterization was not the primary focus, studies lacking a clear description or implementation details, studies with insufficient methodological quality or inadequate control groups, studies conducted in outpatient, primary care, and long-term care settings, and studies published before 2000 due to potential changes in healthcare systems.

### Information sources

We extensively conducted a comprehensive literature search in the following databases until August 8, 2024: Embase, PubMed, Google Scholar, Scopus, Cochrane Library, and Web of Science^20^. Moreover, we reviewed reference lists of the studies included in our analysis to identify any further relevant literature.

### Search strategy

We developed a customized search strategy^21^ for each database to optimize the identification of relevant studies. Table 1 provides a detailed description of the search strategies.

**Table 1:** Search strategy for each database.

database	Search strategy
**Web of Science** (Filters*: English; Article)**:**	((TI=(Catheter* OR indwelling urinary catheter* OR Catheter* Urinary OR Urinary Catheter* OR Catheter*, Ureteral OR Ureteral Catheter* OR Catheter*, Urethral OR Antimicrobial catheters OR Coated catheter* OR Silver alloy catheter* OR Antiseptic catheter* OR Foley catheter*)) AND TI=(Urinary Tract Infect* OR Infect*, Urinary Tract OR Tract Infect*, Urinary OR UTI OR CAUTI OR Catheter care OR Catheter maintenance OR catheter care protocols OR aseptic insertion techniques OR antimicrobial coating)) NOT TI=(Systematic review OR review OR Meta-analysis)
**Scopus** (Filters*: English; Article)**:**	TITLE (catheter* OR (indwelling AND urinary AND catheter*) OR (catheter* AND urinary) OR (urinary AND catheter*) OR (catheter*, AND ureteral) OR (ureteral AND catheter*) OR (catheter*, AND urethral) OR (antimicrobial AND catheter*) OR (coated AND catheter*) OR (silver AND alloy AND catheter*) OR (antiseptic AND catheter*) OR (foley AND catheter*)) AND TITLE ((urinary AND tract AND infect*) OR (infect*, AND urinary AND tract) OR (tract AND infect*, AND urinary) OR uti OR cauti OR (catheter AND care) OR (catheter AND maintenance) OR (catheter AND care AND protocol) OR (aseptic AND insertion AND techniques) OR (antimicrobial AND coating)) AND NOT TITLE ((systematic AND review) OR review OR meta-analysis) AND PUBYEAR > 1999 AND PUBYEAR < 2025
**PubMed** (Filters*: English)**:**	((Catheter*[Title] OR indwelling urinary catheter*[Title] OR Catheter* Urinary[Title] OR Urinary Catheter*[Title] OR Catheter*, Ureteral[Title] OR Ureteral Catheter*[Title] OR Catheter*, Urethral[Title] OR Antimicrobial catheter*[Title] OR Coated catheter*[Title] OR Silver alloy catheter*[Title] OR Antiseptic catheter*[Title] OR Foley catheter*[Title]) AND (Urinary Tract Infect*[Title] OR Infect*, Urinary Tract[Title] OR Tract Infect*, Urinary[Title] OR UTI[Title] OR CAUTI[Title] OR Catheter care[Title] OR Catheter maintenance[Title] OR catheter care protocol*[Title] OR aseptic insertion technique*[Title] OR antimicrobial coating[Title])) NOT (Systematic review[Title] OR review[Title] OR Meta-analysis[Title])
**EMBASE** (Filters*: English; Article)**:**	(catheter*:ti OR ‘indwelling urinary catheter*’:ti OR ‘catheter* urinary’:ti OR ‘urinary catheter*’:ti OR ‘catheter*, ureteral’:ti OR ‘ureteral catheter*’:ti OR ‘catheter*, urethral’:ti OR ‘antimicrobial catheter*’:ti OR ‘coated catheter*’:ti OR ‘silver alloy catheter*’:ti OR ‘antiseptic catheter*’:ti OR ‘foley catheter*’:ti) AND (‘urinary tract infect*’:ti OR ‘infect*, urinary tract’:ti OR ‘tract infect*, urinary’:ti OR uti:ti OR cauti:ti OR ‘catheter care’:ti OR ‘catheter maintenance’:ti OR ‘catheter care protocol*’:ti OR ‘aseptic insertion techniques’:ti OR ‘antimicrobial coating’:ti) NOT (‘systematic review’:ti OR review:ti OR ‘meta analysis’:ti) AND [2000-2024]/py
**Cochrane Central Register of Controlled Trials** (Filters*: English; Trials)**:**	(Catheter* OR indwelling urinary catheter* OR Catheter* Urinary OR Urinary Catheter* OR Catheter*, Ureteral OR Ureteral Catheter* OR Catheter*, Urethral OR Antimicrobial catheters OR Coated catheter* OR Silver alloy catheter* OR Antiseptic catheter* OR Foley catheter*):ti AND (Urinary Tract Infect* OR Infect*, Urinary Tract OR Tract Infect*, Urinary OR UTI OR CAUTI OR Catheter care OR Catheter maintenance OR catheter care protocol* OR aseptic insertion techniques OR antimicrobial coating):ti NOT (Systematic review OR review OR Meta-analysis):ti
**Google Scholar** (Filters: None)**:**	(Catheter* OR indwelling urinary catheter* OR Catheter* Urinary OR Urinary Catheter* OR Catheter*, Ureteral OR Ureteral Catheter* OR Catheter*, Urethral OR Antimicrobial catheters OR Coated catheter* OR Silver alloy catheter* OR Antiseptic catheter* OR Foley catheter*) AND (Urinary Tract Infect* OR Infect*, Urinary Tract OR Tract Infect*, Urinary OR UTI OR CAUTI OR Catheter care OR Catheter maintenance OR catheter care protocols OR aseptic insertion techniques OR antimicrobial coating) NOT (Systematic review OR review OR Meta-analysis) *

All databases were searched on September 3, 2024. After retrieving search results, filters were applied using the filter panel on the results page.

### Selection process

**Figure 1:**
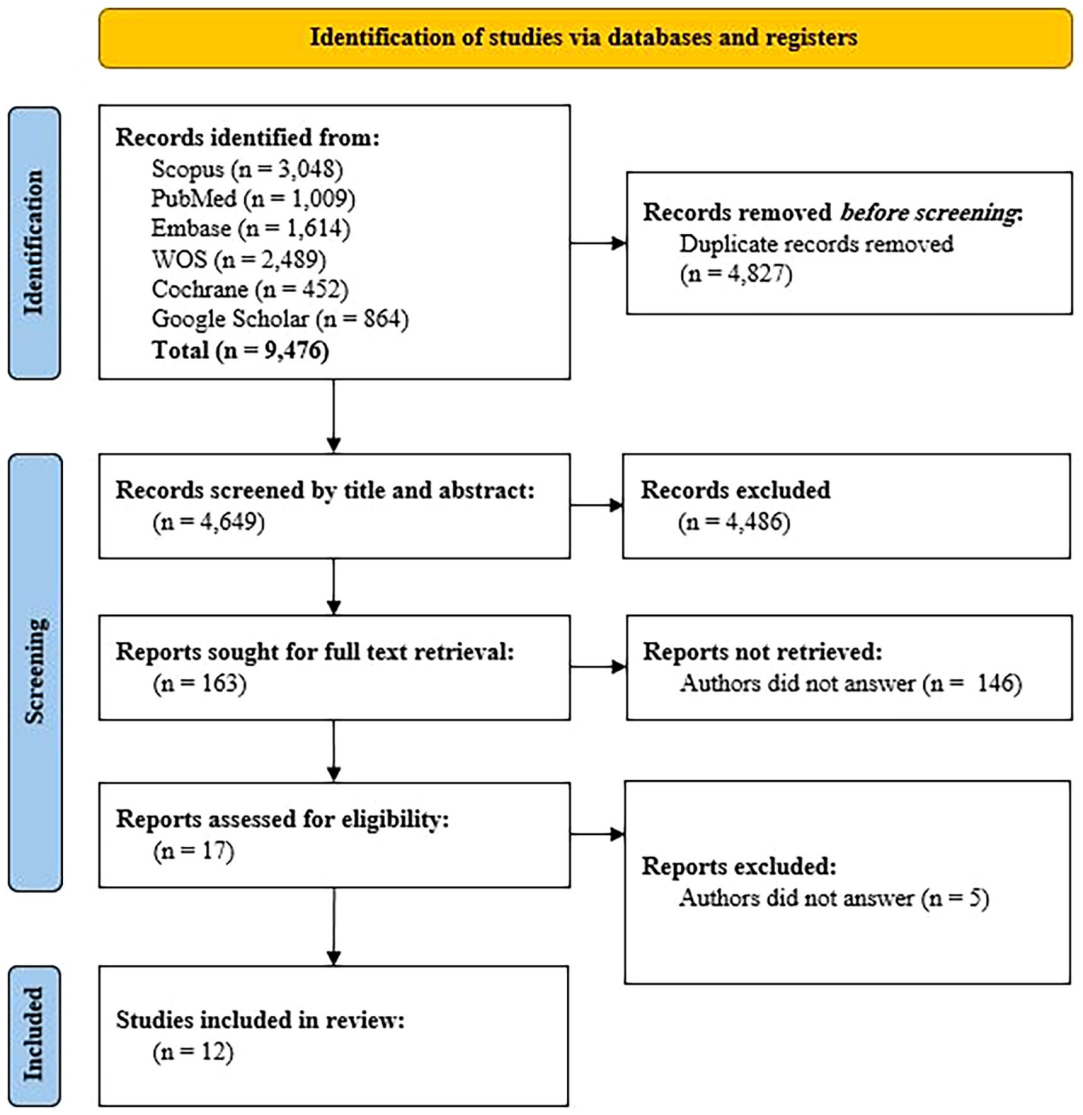
Screening process flowchart.

Search results were imported into EndNote Desktop, and duplicates were removed. Two researchers independently assessed titles and abstracts based on predetermined eligibility criteria. Disagreements in study selection were resolved through full-text review, involving a third researcher if necessary. Attempts were made to acquire inaccessible articles and unpublished data by contacting corresponding authors. The study selection process was visually depicted using a PRISMA 2020 flow diagram^18^(Figure 1).

### Data collection process

A data extraction form was created to ensure consistent data collection, with a calibration exercise performed to compare completed forms for the three main articles. Subsequently, two authors independently extracted data for all studies. Any discrepancies were resolved through discussions with a third author. Extracted data included the following information: author(s), year, country, objective, type of study, inclusion/exclusion criteria, sample size, intervention group, comparison/control group, outcome, adverse events.

### Risk of bias assessment

To evaluate the quality of the studies, we employed two tools:

1. CASP Standard Checklist uses 10-12 items to evaluate the quality of a paper. This checklist evaluates the strengths and weaknesses of the articles, the quality of the study design, and the applicability of studies. The precise checklist was applied according to the type of study, with each item scored as “Yes” (1 point), “No” (0 points), and “Can’t tell” (0 points). Two independent reviewers assessed the articles’ quality separately, with discrepancies resolved through group discussion. Articles with scores of ≥ 75% of the total score were classified as high quality, articles with scores of 25–75% of the total score were classified as moderate quality, and articles with scores of <25% of the total score were classified as poor quality.
2. Revised Cochrane Risk-of-Bias (RoB 2) assesses potential bias in randomized trials across five domains: randomization process, deviations from intended interventions, missing outcome data, outcome measurement, and reporting bias^22^ (Figure 2).
  **Figure 2:**
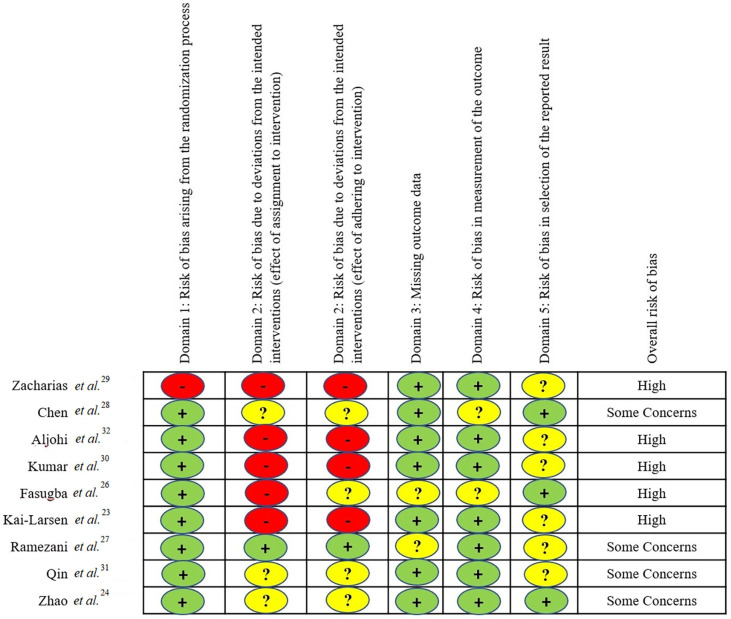
Risk of bias assessment for randomized control trials.

### Data synthesis and analysis

A descriptive and qualitative synthesis of the study results was performed for all included studies, with findings presented in both narrative and tabular formats. A meta-analysis was not conducted due to the substantial heterogeneity observed among the retrieved articles, including variations in study design, population characteristics, types of interventions, and outcome measures. Moreover, a high risk of publication bias was identified, which could compromise the validity of a pooled effect estimate. Consequently, a narrative synthesis was deemed the most appropriate and methodologically sound approach for summarizing the evidence gathered.

## RESULTS

### Literature search

Initial database searches identified 9,476 articles. After removal of 4,827 duplicates, 4,649 titles and abstracts remained, resulting in 163 full texts for further review. A total of 17 eligible articles were selected for quality and risk of bias assessment, of which 12 were included in the final analysis (Figure 1).

### Study characteristics

The included studies were conducted in various countries (Australia, Iran, India, Taiwan, the U.S., Korea, China, and Saudi Arabia) from 2009 to 2024. They involved 60-1642 participants aged 18-78 years. While one study included only male^23^ and three included only female^24-26^ participants, most studies included both sexes^27-33^. One study did not specify the gender distribution^34^. A total of 11 studies employed two intervention and comparison/control groups^23-33^, whereas one study used four experimental groups^34^.

Of the 12 studies included, seven specifically targeted critically ill patients or were conducted in intensive care unit (ICU) settings. These included studies by Ramezani *et al*.^28^ (ICU patients with GCS ≤ 9), Kai-Larsen *et al*.^24^ (patients in ICU, surgical, or urology units), Zhao *et al*.^25^ (critically ill ICU patients), Chen *et al*.^29^ (respiratory ICU), Galiczewski and Shurpin^23^ (medical ICU), Jeong *et al.* ^34^ (patients in emergency, medical, or neurosurgical ICUs), and Qin *et al*.^32^ (comatose ICU patients with long-term catheterization). The remaining five studies were conducted in general hospital or ward settings without specific focus on intensive care. These included studies by Fasugba *et al*.^27^ (hospitalized patients across wards), Sarani *et al.* ^26^ (neurology ward), Zacharias *et al*.^30^ (neurosurgical patients), Kumar *et al*.^31^ (general hospitalized population), and Aljohi *et al*.^33^ (adult inpatients with no ICU specification). This distinction is important for interpreting intervention effectiveness, as ICU patients often have higher infection risks and require more intensive monitoring (Table 2).

**Table 2:** Characteristics of included studies

Article	Country	Objective	Type of Study	Inclusion/Exclusion Criteria	Sample size	Intervention Group				Comparison/Control Group			Outcome	Adverse effects	Article’s quality
						N, Gender (M/F)	Age (Mean±SD)	Interventions	Duration	N, Gender (M/F)	Age (Mean±SD)	Comparison/Control			
Fasugba *et al.* ^27^	Australia	Effect of Chlorhexidine on reducing catheter-associated urinary tract infections	Randomized controlled trial	**Inclusion criteria:** all hospitalized patients requiring a urinary catheter **Exclusion criteria:** patients younger than 2 years, had an allergy, had contraindication or other medical reason preventing the use of chlorhexidine, had the catheter inserted in the theatre, did not have the catheter insertion date documented, required in-and-out or suprapubic catheterization, had symptoms and signs suggestive of UTI at the time of catheter insertion, or were undergoing treatment for UTI	1642	**M:** 325 (34%) **F:** 620 (66%)	50 (32–76)	0·1% chlorhexidine solution	7 days	**M:** 368 (53%) **F:** 329 (47%)	78 (69–87)	0·9% normal saline	The intervention decreased the incidence of catheter-associated UTI (94%).	No adverse events	Moderate
Ramezani *et al.* ^28^	Iran	Evaluating the Potential of a New Low-Profile Urinary Catheter in Preventing Catheter-Associated Urinary Tract Infections	Prospective randomized blinded clinical trial	**Inclusion criteria:** all patients who were candidates for urinary catheterization and were admitted directly from the Emergency Department to the ICU, with a ≤ 9 score of consciousness on the Glasgow Coma Scale (GCS), and 18 years or older, patients had no recent history of UTI and use of aminoglycoside medications, and no history of diabetes, chronic kidney disease (CKD), and immune system diseases. **Exclusion criteria:** any planned or unplanned removal of the patient’s urinary catheter before 3 days, patient death, or transfer outside the ICU during the study.	80	**M:** 16 (40.0%) **F:** 24 (60.0%)	49.78 (19.91)	Low-profile catheter	5 days	**M:** 21 (52.5%) **F:** 19 (47.5%)	50.48 (18.67)	Standard Foley catheter	Results show the effectiveness of low-profile urinary catheters compared to standard Foley catheters in preventing CAUTI in patients.	Not mentioned	Moderate
Kai-Larsen *et al.* ^24^	India	Effect of Foley catheter with noble metal alloy coating for preventing catheter-associated urinary tract infections	Randomized controlled trial	**Inclusion criteria:** adults aged > 18 years requiring urethral catheterization (closed drainage system) for ≥ 48 h, provided they were admitted to the hospital for urology, surgery, and an ICU stay. **Exclusion criteria:** pregnant or breastfeeding patients, receiving antibiotic treatment for a UTI or catheterization, had a latex allergy, or had undergone previous urinary tract surgery likely to interfere with the study results.	1000	**F:** 235 (31)	48 (15)	NMA^a^-coated BIP Foley Catheter	2 days	**F:** 101 (40)	47 (15)	Non-coated control catheter	Incidence of symptomatic CAUTI was reduced by 69% in the BIP Foley Catheter group compared with control.	Lower incidence of adverse events in NMA-coated BIP Foley catheter compared to the uncoated catheter	Moderate
Chen *et al.* ^29^	Taiwan	Using a criteria-based reminder to reduce use of indwelling urinary catheters and decrease urinary tract infections	Randomized control trial	**Inclusion criteria:** Patients with an indwelling urinary catheter in place at the time of admission or during their stay in the RICUs from April to November 2008. **Exclusion criteria:** Patients without an indwelling urinary catheter or RICU stay for less than 2 days.	278	**M:** 113 (76.9) **F:** 34 (23.1)	78 (10.5)	Use of a criteria-based reminder for catheter removal	7 days	**M:** 94 (71.8) **F:** 37 (28.2)	77 (12.7)	Control group (no reminder)	The reminder intervention reduced the incidence of catheter-associated infections by 48% in the intervention group compared with the control group.	No major complications were detected during the study period.	Moderate
Galiczewski and Shurpin ^23^	U.S	Intervention to improve catheter-associated urinary tract infection rate in a medical intensive care unit	Case-control study	**Inclusion criteria:** patients ≥18 years of age, patients admitted or transferred to the medical ICU, required an indwelling urinary catheter, and catheter was placed in the medical ICU. **Exclusion criteria:** patients <18 years of age and patients with a urinary catheter placed in another unit of the hospital prior to being transferred or admitted to the medical ICU.	140	**M:** 66 (56.1%)	66.15 (16.58)	Education session	30 minutes	**M:** 74 (56.8%)	67.03 (16.35)	Standard care	CAUTI rates decreased from 2.24 to 0 per 1000 catheter days.	Not mentioned	Moderate
Zacharias *et al.* ^30^	India	The effect of amikacin sulfate bladder wash on catheter-associated urinary tract infection in neurosurgical patients	Prospective randomized controlled	Urine samples were sent within 24 hours for culture and sensitivity (C/S); in case of positive C/S, patients were excluded from the study	60	**M:** 16 (53) **F:** 14 (47)	19–65 (38)	Amikacin bladder wash	Twice daily	**M:** 16 (53) **F:** 14 (47)	18–68 (42.5)	Control group	Amikacin sulfate bladder wash was effective in preventing CAUTI.	Not mentioned	Moderate
Jeong *et al.* ^34^	Korea	Comparison of Catheter-associated Urinary Tract Infection Rates by Perineal Care Agents in Intensive Care Units	Experimental study	**Inclusion criteria**: patients newly admitted to an emergency, medical or neurosurgical ICU at a National University Hospital in Korea between April 1 and July 31, 2008; were female over 20 years of age; no diagnosis of a urinary tract infection, that is, negative urine cultures prior to the study; without an indwelling catheter when admitted to the ICU or had an indwelling catheter inserted in the emergency room in the study hospital within 12 hours before admittance to an ICU, and who had baseline urine culture; and kept the indwelling catheter in situ at least 2 days after insertion.	97	**-**	**Group 1:** 64.1 (13.3) **Group 2:** 54.3 (14.5) **Group 3:** 61.5 (17.3) **Group 4:** 60.8 (15.9)	**Group 1:** soap-and-water, **Group 2:** skin cleansing foam, **Group 3:** 10% povidone iodine solution, **Group 4:** normal saline	4 weeks	**-**	-	-	The type of perineal care does not influence CAUTI incidence.	Not mentioned	Moderate
Zhao *et al.* ^25^	China	Prevention of urinary tract infection using a silver alloy hydrogel-coated catheter in critically ill patients	Randomized controlled study	**Inclusion criteria:** critically ill patients aged ≥18 years in the ICU who required indwelling catheterization. **Exclusion criteria:** estimated time of indwelling catheter <14 days; long-term use of glucocorticoids or immunosuppressive drugs; malignancy; hematological diseases; autoimmune diseases or immunodeficiency; history of structural urinary tract diseases (like prostatic hyperplasia, urinary calculus, urethral malformation, etc.); patients with indwelling catheterization for >48 h before admission; urinary tract infection or latent infection before admission to ICU; and acute or chronic renal function injury.	132	**F:** 28.0 (43.8)	60.0 (18.7)	Silver alloy hydrogel-coated catheter	14 days	**F:** 30.0 (44.1)	64.6 (17.5)	Conventional siliconized latex Foley catheter	SAH catheters can reduce the incidence of CAUTIs, compared with conventional siliconized latex Foley catheters	Not mentioned	Moderate
Sarani *et al.* ^26^	Iran	Comparison of the Effect of Perineal Care with Normal Saline and 2% Chlorhexidine Solution on the Rate of Catheter-Associated Urinary Tract Infection	Quasi-experimental study	**Inclusion criteria:** patients aged 18 to 55 years having a GCS of 8 or lower, no history of UTI, no congenital urinary tract disorders, no malignancy and immune deficiency, no diabetes, no vaginal infection, no genital sores, and no sensitivity to chlorhexidine solution. **Exclusion criteria:** positive urine culture on the first day, patient death, patient transferred to other wards before completing the study, catheter removal before the seventh day, abdominal or pelvic surgery, allergy to chlorhexidine solution, and wound formation in the genital area.	60	**F:** 30	40.17 (11.52)	2% Chlorhexidine	Twice a day for 7 days	**F:** 30	38.71 (13.38)	Normal saline	Seven days after the intervention, the incidence of UTIs was significantly lower in the chlorhexidine group (13.3%) than in the normal saline group	Not mentioned	Moderate
Kumar *et al.* ^31^	India	Role of neomycin polymyxin sulfate solution bladder wash in preventing catheter-associated urinary tract infection	Prospective randomized controlled study	All patients that catheterized within 24 hours were included. **Exclusion criteria:** Patient with history of pregnancy, immunocompromised patients (AIDS, chemotherapy), diabetes, recurrent UTI, renal insufficiency, use of antibiotics within the previous 7 days and catheter removed before the 3rd day	100	**M:** 30 (60) **F:** 20 (40)	38 (12.6)	Neomycin and Polymyxin Sulphate solution bladder wash	7 days	**M:** 35 (70) **F:** 15 (30)	39.4 (13.2)	Normal saline	Significant reduction in CAUTI incidence in neomycin/polymyxin test group in comparison with the normal saline irrigated control group.	Not mentioned	Moderate
Qin *et al.* ^32^	China	Efficacy of expanded periurethral cleansing in reducing catheter-associated urinary tract infection in comatose patients	Randomized controlled clinical trial	**Inclusion criteria:** adult patients (≥ 18 years), Glasgow Coma Scale score ≤ 8, without preexisting urological diseases/abnormalities or obstetric/gynecological abnormalities, and requiring a urinary catheter for at least 10 days. **Exclusion criteria:** patients with a history of allergic reactions to povidone-iodine or who had positive baseline urine cultures (urine specimens were collected immediately after catheterization).	446	**M:** 93 **F:** 132	49.93 (10.31)	Expanded periurethral cleansing protocol	10 days	**M:** 116 **F:** 105	50.04 (11.13)	Usual periurethral cleansing protocol	Expanded periurethral cleansing could reduce the incidence of CAUTI.	Not mentioned	Moderate
Aljohi *et al.* ^33^	Saudi Arabia	The efficacy of noble metal alloy urinary catheters in reducing catheter-associated urinary tract infection	Randomized study	**Inclusion criteria:** adult patients (≥18 years), no UTI, requiring a urinary catheter for at least 3 days and using a closed drainage system. **Exclusion criteria:** children, patients with UTIs, patients using open drainage system, urinary tract congenital abnormalities or obstetric/gynecological abnormalities, and patients with duration of urinary catheter <3 days.	60	**M:** 16 (53) **F:** 14 (47)	43.9 (21.7)	Latex noble metal alloy catheters	3 days	**M:** 14 (47) **F:** 16 (53)	58.4 (19.5)	Conventional siliconized latex Foley catheters	A 90% relative risk reduction in the rate of CAUTI was observed with the noble metal alloy catheter compared to the standard catheter	No adverse events related to any of the used catheters were recorded	Moderate


^a^Noble Metal Alloy.

### Interventions to prevent catheter-associated urinary tract infections

The twelve included studies examined diverse approaches to prevent CAUTIs. To enhance conceptual clarity and facilitate interpretation, the interventions were categorized into three main groups: Catheter-Integrated Technological Innovations, Adjunctive Antimicrobial and Antiseptic Strategies, and Healthcare System and Practice-Based Interventions. Each category represents a different level of intervention, ranging from changes in device design to procedural and system-level enhancements (Table 3).

**Table 3:** Comparative effectiveness of interventions for preventing catheter-associated urinary tract infections (CAUTIs).

Intervention category	Article	Intervention type	CAUTI Reduction	Effect size / CI	Statistical significance
**Catheter-integrated**	Ramezani *et al.* ^28^	Low-profile catheter	Effective compared with standard Foley	Not specified	p = 0.007
	Kai-Larsen *et al.* ^24^	NMA-coated Foley catheter	69% symptomatic CAUTI reduction	IRR = 0.31; 95% CI: 0.21–0.46	p < 0.001
	Aljohi *et al.* ^33^	Noble metal alloy catheter	90% CAUTI reduction	Not specified	p = 0.006
	Zhao *et al.* ^25^	Silver alloy hydrogel-coated catheter	Significant reduction in CAUTIs	Not specified	p < 0.05
**Antimicrobial/Antiseptic strategies**	Fasugba *et al.* ^27^	0.1% Chlorhexidine cleansing	94% reduction in CAUTI	IR = 0.06; 95% CI: 0.01-0.32	p = 0.00080
	Sarani *et al.* ^26^	2% Chlorhexidine vs. saline	76.7% to 13.3% infection rate	Not specified	p = 0.001
	Zacharias *et al.* ^30^	Amikacin bladder wash	Effective in preventing CAUTI	Not specified	p < 0.001
	Kumar *et al.* ^31^	Neomycin/polymyxin bladder wash	Significant reduction in CAUTI	Not specified	p < 0.05
	Qin *et al.* ^32^	Expanded periurethral cleansing	Reducing the incidence of CAUTI	Not specified	p < 0.05
	Jeong *et al.* ^34^	Soap and water, skin cleansing foam, 10% povidone-iodine solution, and normal saline	No significant difference	Not specified	P > 0.05
**System/Practice-based**	Chen *et al.* ^29^	Reminder protocol	48% CAUTI reduction	RR = 0.52; 95% CI: 0.32–0.86	p = 0.009
	Galiczewski and Shurpin^23^	Education session	2.24 to 0 CAUTIs per 1,000 catheter days, but not significant	Not specified	p = 0.253

### Catheter-integrated technological innovations

This category includes interventions involving structural or material modifications to catheters aimed at reducing bacterial colonization and biofilm formation, key mechanisms in CAUTIs pathogenesis.

One study found that low-profile catheters significantly reduced CAUTI rates compared with standard Foley catheters, likely due to less traumatic insertion and better urine drainage^28^. Two studies evaluated noble metal alloy-coated catheters (incorporating silver, gold, and palladium), with one reporting a 69% reduction in symptomatic CAUTIs^24^ and another showing a 90% relative risk reduction in critically ill patients^33^. Similarly, silver alloy hydrogel-coated catheters, which combine antimicrobial properties with a lubricating surface, significantly reduced infection rates compared with conventional latex catheters^25^. These findings highlight the importance of catheter material in reducing infection, particularly in high-risk ICU settings.

### Adjunctive antimicrobial and antiseptic strategies

This category encompasses supportive interventions used alongside standard catheterization, primarily focused on reducing the microbial burden with localized disinfection or bladder irrigation.

Chlorhexidine-based cleansing was assessed by two studies^26,27^, both showing a significantly lower incidence of CAUTIs compared with normal saline, reinforcing chlorhexidine’s efficacy as a broad-spectrum antiseptic. Two studies evaluated bladder irrigation with antimicrobial agents such as amikacin or a neomycin–polymyxin combination, which reduced infection rates by directly flushing pathogens from the bladder^30,31^. Findings on perineal cleansing protocols were inconsistent. One study compared four cleansing agents and found no significant differences^34^, whereas another showed that more frequent and thorough periurethral cleaning significantly lowered infection rates^32^.

Together, these studies highlight that both the type and frequency of cleansing are critical components in infection prevention.

### Healthcare system and practice-based interventions

These interventions target care processes and staff behaviors rather than directly modifying catheter or patient care. They are designed to improve decision-making regarding catheter use and ensure adherence to best practices.

In one study, a criteria-based reminder system implemented as an electronic or paper-based tool prompted clinicians to review the necessity of indwelling urinary catheters, resulting in a 48% reduction in CAUTI rates by reducing unnecessary catheterization days^29^. Such reminders align with clinical guidelines recommending daily catheter need assessment. Another study introduced a 30-minute educational session for ICU staff to reinforce evidence-based practices for catheter insertion, maintenance, and removal. This intervention resulted in a drop in CAUTI rates from 2.24 to 0 per 1,000 catheter days^23^. The authors suggested that even brief training, when consistently applied, can have a substantial impact.

These results suggest the effectiveness of behavioral and process-oriented strategies as powerful complements to technical interventions, especially in settings with high device utilization.

### Outcomes

Of the twelve studies reviewed, eleven showed the effectiveness of various interventions in preventing CAUTIs. One study, however, reported no statistically significant results (Table 2).

A preventive intervention provided a 74% reduction in asymptomatic catheter-associated bacteriuria (incidence ratio 0.26, 95%CI 0.08–0.86, p = 0.026) and a 94% reduction in CAUTI incidence (incidence ratio 0.06, 95%CI 0.01–0.32, p = 0.00080)^27^.

While standard Foley catheters showed a trend toward more positive urine cultures on day 5 compared with a low-profile catheter, logistic regression analysis indicated no statistically significant difference in CAUTI risk between them (odds ratio 3.22; p = 0.208)^28^.

BIP Foley catheter reduced in 69% the symptomatic CAUTI incidence compared with the control (6.5 vs. 20.8 CAUTI/1000 catheter days, incidence rate ratio of 0.31, 95%CI 0.21–0.46, p < 0.001), with the most significant reduction observed between days 3 and 11 post-catheterization^24^. A criterion-based reminder intervention effectively reduced catheter-associated infections by 48% (relative risk, 0.52; 95%CI, 0.32-0.86; p = 0.009) compared with routine care^29^.

One study reported a significant reduction in CAUTI rates from 2.24 to 0 per 1000 catheter days^23^. Another study found that none of the participants in the amikacin bladder wash group developed CAUTI compared to 40% in the control group (Fisher’s exact test, p < 0.001)^30^.

However, the type of perineal care did not affect CAUTI rates. Cumulative incidence of CAUTIs per 100 catheter days was 3.18 episodes for one week, 3.31 for two weeks, and 3.04 for four weeks. We found no statistically significant differences in the hazard ratios for CAUTIs associated with any perineal care factor, including soap and water, at one, two, or four weeks after the start of perineal care^34^.

A silver alloy hydrogel (SAH) catheter significantly reduced the positivity rates for urinary white blood cells, positive urine cultures, and CAUTIs compared with a conventional catheter on days 7 and 10 post-catheterization^25^. Chlorhexidine solution significantly reduced UTI incidence compared with normal saline (13.3% vs. 76.7%, p = 0.001)^26^. Similarly, neomycin/polymyxin solution significantly reduced CAUTI incidence compared with normal saline irrigation^31^.

One study showed that CAUTI incidences in the experimental and control groups were (5/225, 2.22% vs. 7/221, 3.17%, p = 0.54), (12/225, 5.33% vs. 18/221, 8.14%, p = 0.24), and (23/225, 10.22% vs. 47/221, 21.27%, p = 0.001) on days 3, 7, and 10, respectively. Bacterial CAUTI and fungal CAUTI incidences in the two groups were (11/225, 4.89% vs. 24/221, 10.86%, p = 0.02) and (10/225, 4.44% vs. 14/221, 6.33%, p = 0.38), respectively. Polymicrobial CAUTI incidences in the two groups were 2/225 (0.89%) and 9/221 (4.07%), respectively (p = 0.03)^32^. Use of noble metal alloy catheter resulted in a 90% reduction in CAUTI rate compared with the standard catheter (10 vs. 1 cases, p = 0.006)^33^.

### Adverse effects

Four studies provided information on adverse effects associated with various interventions. Fasugba *et al.* ^27^ and Aljohi *et al.* ^33^ reported no adverse events. Kai-Larsen *et al.* ^24^ found no serious adverse events related to either catheter type. Few patients in the NMA-coated BIP Foley catheter group experienced adverse events compared with the control group (21.6% vs. 48.4%; p = 0.001). Chen *et al.* ^29^ detected no major complications during the study period.

## DISCUSSION

Our study investigated interventions aimed at preventing catheter-associated urinary tract infections. Across the twelve included studies, most interventions effectively reduced CAUTI incidence.

A key distinction among the studies was the patient care setting. Seven studies focused on critically ill or intensive care unit (ICU) populations^23-25,28,29,32,34^ where prolonged catheter use, immunosuppression, mechanical ventilation, and reduced mobility increased infection risks. ICU patients often have multiple comorbidities, undergo frequent invasive procedures, and face prolonged exposure to hospital pathogens, all of which increase the risk of catheter colonization and infection. Additionally, ICU protocols often involve extended catheterization for monitoring fluid status or urinary output, making preventive strategies even more essential. In contrast, studies conducted in general hospital wards showed the effectiveness of CAUTI interventions in routine care settings^26,27,30,31,33^ where patients typically have shorter hospital stays, lower acuity levels, and fewer risk factors for infection. Although catheterization is still required, these patients often benefit from earlier catheter removal and experience fewer complications. This distinction underscores the importance of tailoring CAUTI prevention strategies to specific patient populations and care environments.

Our study found a significant reduction in CAUTIs incidence during the intervention period using 0.1% or 0.2% chlorhexidine solution before catheterization, suggesting its effectiveness in preventing CAUTIs. This aligns with previous studies, such as a randomized controlled trial (RCT) reporting a 26% reduction in bacteriuria among males who received daily chlorhexidine baths, highlighting its potential in reducing catheter-associated bacteriuria^35^. Other studies have shown that perineal or catheter irrigation with chlorhexidine significantly decreases UTI incidence compared with normal saline or povidone-iodine^26,36^.

Some RCTs reported no significant effect of chlorhexidine, likely due to methodological limitations. These include small sample sizes, lack of power analysis, and limited generalizability, such as young obstetric patients^37-39^. Additionally, studies incorporating chlorhexidine into multi-agent cleaning protocols struggled to isolate its specific impact.

Another key finding of the present study relates to the influence of catheter type on CAUTIs incidence. Several studies assessing advanced catheter materials, including low-profile catheters, NMA-coated BIP Foley catheters, silver alloy hydrogel (SAH) catheters, and latex noble metal alloy catheters showed reductions in CAUTI rates, emphasizing the role of catheter surface modifications in infection prevention.

Coated catheters, designed to inhibit biofilm formation and bacterial adhesion, outperformed standard silicone and latex catheters. Notably, the SAH catheter—coated with gold, silver, and palladium—creates a microfluidic surface that blocks retrograde bacterial invasion without releasing toxic substances^40^. A large RCT involving 27,878 patients reported a 32% reduction in urinary tract infections, a 21% decrease in CAUTIs, a decline in secondary bloodstream infections, and a 44% reduction in contaminating microorganisms^41^. Similarly, the Bactiguard infection protection (BIP) Foley catheter, with its non-releasing noble metal coating, effectively reduced CAUTI rates across various clinical settings, including ICUs, burn units, and rehabilitation centers^33,40,42^.

Our study showed that a reminder sheet with appropriate indications for removal of indwelling urinary catheters reduced the use of such catheters and consequently decreased CAUTIs incidence in patients with respiratory disease. Minimizing catheterization duration is a crucial strategy for preventing CAUTIs^43,44^. The Healthcare Infection Control Practices Advisory Committee recommends using reminder systems to assess the need for continued catheterization^43^. Meddings *et al.* ^45^ found that reminder interventions decreased mean catheterization duration by 37%, resulting in 2.61 fewer days of catheterization per patient in an intervention group compared with control groups. However, two studies reported no significant effect on UTI rates^46,47^.

We also explored various preventive methods for reducing catheter-related infections, including bladder irrigation and catheter cleaning techniques. Methods using amikacin, neomycin, and polymyxin sulfate significantly reduced catheter-related infections^30-32^, whereas skin cleansing foam, povidone-iodine, soap and water, and normal saline showed no notable differences in infection rates^34^, highlighting the varying effectiveness of cleaning and irrigation agents.

International guidelines on CAUTI management and prevention recommend cleansing the periurethral area^48^. Many RCT have shown that periurethral cleansing reduces the risk of bacterial colonization in the perineal area, thereby reducing the likelihood of opportunistic pathogens entering the urinary tract^27,38,49^.

The effect of expanded periurethral cleansing on reducing CAUTI can be attributed to several factors:

- Microbial reduction: Human skin harbors pathogenic microorganisms, particularly in the periurethral and anal regions where sweat and sebaceous glands create a humid environment conducive to microbial growth^32^ ^,^ ^50^. Expanded periurethral cleansing removes these microorganisms, reducing the risk of infection.
- Immune system support: Comatose patients with urinary catheters often experience immune dysfunction^51^, increasing CAUTI susceptibility. Expanded periurethral cleansing can reduce this burden on the weakened immune system.
- Fecal contamination prevention: Comatose patients are prone to fecal incontinence which can introduce intestinal microorganisms to the periurethral area^52^. Expanded periurethral cleansing reduces microbial colonization around the anal skin, limiting pathogen entry into the urinary tract^32^.

The applicability of CAUTI prevention strategies in low-resource settings is a crucial consideration. While highly effective interventions like silver-coated or noble metal alloy catheters reduce infection rates, their high cost and limited availability may render them impractical in budget-constrained hospitals. However, several low-cost strategies showed strong results and are well-suited for these settings. For example, using 0.1–2% chlorhexidine for cleansing, implementing simple reminder systems to prompt catheter removal, and providing short training sessions for nurses effectively reduced infection rates. These interventions require no advanced equipment and can be integrated into routine care with basic training, making them feasible in low-source environments. Further research is needed to evaluate the cost-effectiveness and implementation challenges of these strategies in low- and middle-income countries, but existing evidence suggests significant improvements can be made without major infrastructure investments.

While this review has identified several promising strategies, methodological limitations must be critically considered to contextualize these findings. The absence of a meta-analysis due to substantial heterogeneity in study designs, patient populations, intervention types, and outcome measures precluded pooling data into a single effect size. This heterogeneity weakens the strength of recommendations, particularly under frameworks like GRADE, which prioritize quantitative synthesis for robust evidence. Without meta-analytic estimates, precise measurements of intervention effectiveness or subgroup analyses to explore context-specific impacts are unattainable, limiting the generalizability of findings and the ability to make strong, universal clinical recommendations. However, the narrative synthesis still offers valuable practical insights by identifying patterns and contextual factors across studies, supporting evidence-based decision-making tailored to local healthcare settings and patient populations.

While antiseptic protocols, coated catheters, and reminder systems are promising, their implementation requires careful consideration of local resources and context. Future research should focus on improving methodological consistency, standardizing outcome measures, and reporting data to facilitate meta-analyses, thereby enhancing the strength and applicability of recommendations.

### Implications of the study

Our findings have several important implications for clinical practice, infection control policies, and future research. First, the consistent effectiveness of antiseptic solutions—particularly 0.1–0.2% chlorhexidine—highlights the need to standardize their use for catheter site preparation and perineal cleansing in both ICU and general ward settings. Hospitals should consider incorporating chlorhexidine as a routine antiseptic for urinary catheterization procedures. Second, the efficacy of advanced catheter technologies (e.g., silver alloy hydrogel-coated and NMA-coated catheters) suggests that device selection plays a critical role in infection prevention. Health systems may benefit from prioritizing these coated catheters for high-risk populations, particularly in ICUs where the burden of CAUTIs is greatest.

Additionally, system-level interventions such as reminder systems and brief educational programs effectively reduced unnecessary catheter use and CAUTI incidence. These findings support the inclusion of CAUTI-specific protocols in broader hospital quality improvement initiatives. Implementing decision support tools and reinforcing staff training may enhance adherence to catheter management guidelines.

Finally, the varying effectiveness of irrigation and cleansing agents underscores the importance of evidence-based product selection. Not all common practices yield significant benefits. Thus, hospitals should prioritize interventions supported by clinical trial data. Future research should further explore the context-specific effectiveness, cost-efficiency, and implementation barriers of each intervention, especially in resource-limited settings.

### Limitations

A key limitation of this review is the considerable heterogeneity among studies and potential publication bias, which made conducting a meta-analysis unfeasible. This restricts quantitative synthesis of results and diminishes statistical power in evaluating the effectiveness of interventions. Variations in methodological quality across studies may affect the reliability of results and introduce biases that could influence the overall findings. Moreover, restricting the study to English-language publications introduces a risk of language bias, potentially excluding relevant non-English studies. Our focus on adult patients in intensive care settings limits generalizability to other healthcare contexts, such as outpatient or long-term care settings.

Despite efforts to access unpublished studies and data, the study may still be affected by selection and reporting biases as it relies on available literature. Sparse reporting of adverse events is another limitation; few studies provided such data, often noting minimal or no adverse effects, which may underreport potential adverse outcomes associated with the interventions. This lack of detailed safety information hinders comprehensive conclusions about intervention safety profiles and highlights the need for more rigorous adverse effect reporting in future research. Lastly, the exclusion of studies published before 2000, while seeking to maintain relevance to current healthcare practices, may omit valuable historical data that could provide a broader understanding of CAUTI prevention methods. Addressing these limitations in future research would strengthen the applicability and reliability of findings in this field.

## CONCLUSION

This review underscores the effectiveness of implementing multiple strategies to significantly reduce CAUTI rates. The use of chlorhexidine solution as a pre-catheterization antiseptic, the adoption of advanced catheter materials like silver alloy hydrogel and NMA-coated BIP Foley catheters, and the implementation of reminder systems for timely catheter removal and expanded periurethral cleansing practices have all shown promising infection prevention. To further solidify these findings and optimize CAUTI prevention strategies, future research should prioritize large-scale, multicenter randomized controlled trials to validate promising interventions such as chlorhexidine-based cleansing and device-based innovations like silver alloy and noble metal-coated catheters. These studies should include standardized outcome measures and stratify results by patient risk groups and care settings (e.g., ICU vs. general ward). Concurrently, cost-effectiveness analyses are urgently needed to determine the financial feasibility of implementing coated catheters and antimicrobial bladder washes in routine practice. System-based interventions, such as reminder systems and staff education, should also be investigated for long-term sustainability, compliance, and scalability, particularly in low-resource or high-burden settings. Lastly, future studies should incorporate qualitative process evaluations to better understand implementation barriers and facilitators across diverse healthcare environments.

## Data Availability

The complete anonymized dataset supporting the findings of this study is included within the article itself.
